# Effect of Fibre Material and Fibre Roughness on the Pullout Behaviour of Metallic Micro Fibres Embedded in UHPC

**DOI:** 10.3390/ma13143128

**Published:** 2020-07-14

**Authors:** Niels Wiemer, Alexander Wetzel, Maximilian Schleiting, Philipp Krooß, Malte Vollmer, Thomas Niendorf, Stefan Böhm, Bernhard Middendorf

**Affiliations:** 1 Department of Structural Materials and Construction Chemistry, University of Kassel, 34125 Kassel, Germany; alexander.wetzel@uni-kassel.de (A.W.); schleiting@uni-kassel.de (M.S.); middendorf@uni-kassel.de (B.M.); 2 Institute of Material Engineering, University of Kassel, 34125 Kassel, Germany; krooss@uni-kassel.de (P.K.); vollmer@uni-kassel.de (M.V.); niendorf@uni-kassel.de (T.N.); 3 Department for Cutting and Joining Processes, University of Kassel, 34125 Kassel, Germany; s.boehm@uni-kassel.de

**Keywords:** high-performance concrete, fibre reinforcement, shape memory alloys, pullout strength, fibre/matrix bond

## Abstract

The use of micro fibres in Ultra-High-Performance Concrete (UHPC) as reinforcement increases tensile strength and especially improves the post-cracking behaviour. Without using fibres, the dense structure of the concrete matrix results in a brittle failure upon loading. To counteract this behaviour by fibre reinforcement, an optimal bond between fibre and cementitious matrix is essential. For the composite properties not only the initial surfaces of the materials are important, but also the bonding characteristics at the interfacial transition zone (ITZ), which changes upon the joining of both materials. These changes are mainly induced by the bond of cementitious phases on the fibre. In the present work, three fibre types were used: steel fibres with brass coating, stainless-steel fibres as well as nickel-titanium shape memory alloys (SMA). SMA fibres have the ability of “remembering” an imprinted shape (referred to as shape memory effect), triggered by thermal activation or stress, principally providing for superior performance of the fibre-reinforced UHPC. However, previous studies have shown that NiTi-fibres have a much lower bond strength to the concrete matrix than steel fibres, eventually leading to a deterioration of the mechanical properties of the composite. Accordingly, the bond between both materials has to be improved. A possible strategy is to roughen the fibre surfaces to varying degrees by laser treatment. As a result, it can be shown that laser treated fibres are characterised by improved bonding behaviour. In order to determine the bond strength of straight, smooth fibres of different metal alloy compositions, the present study characterized multiple fibres in series with a Compact-Tension-Shear (CTS) device. For critical evaluation, results obtained by these tests are compared with the results of conventional testing procedures, i.e., bending tests employing concrete prisms with fibre reinforcements. The bond behaviour is compared with the results of the flexural strength of prisms (4 × 4 × 16 cm^3^) with fibre reinforcements.

## 1. Introduction

### 1.1. Charateristics of UHPC

Ultra-High-Performance Concrete (UHPC) is a very dense type of concrete being characterised by compressive strengths values between 150 and 250 MPa [1]. Besides high strength, the durability is enhanced as well. Factors contributing to these unique properties are an optimized packing density, a high cement content and a low water content. Silica fume acts as micro-filler and as a further strengthening component due to a pozzolanic reaction with Ca(OH)_2_ from cement hydration. A low water content (water/binder ratio of about 0.20 to 0.25) leads to a low capillary porosity, reducing the permeability of water and other fluids [1,2]. The bond between matrix and aggregate is improved due to the packing density optimisation and the additional calcium silicate hydrate (C-S-H) phases. Polycarboxylatether (PCE) based superplasticizers are used to create the required workability of the fresh concrete.

**Figure 1:**
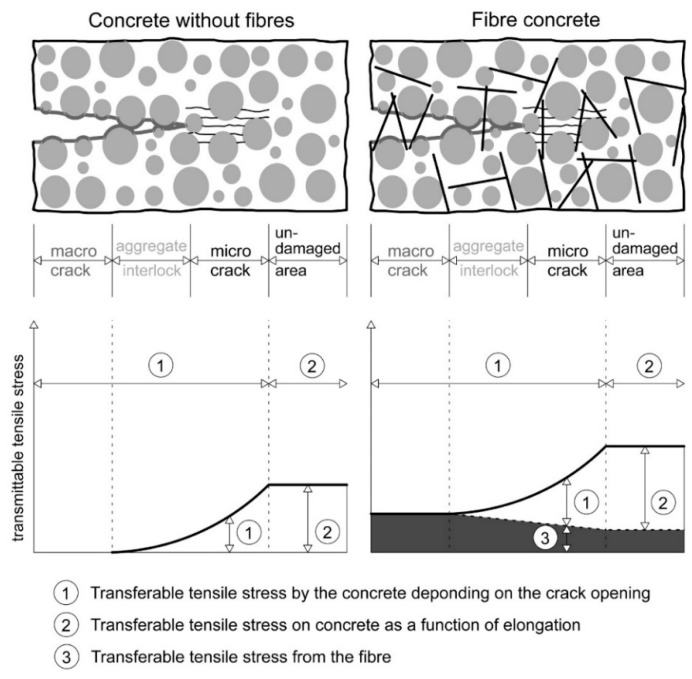
Schematic representation highlighting the fracture mechanical effect of steel fibres in concrete. Functioning of the fibres in the transition from micro to macro cracking (based on [8]).

Due to its high density, UHPC has brittle behaviour upon mechanical load without fibres. After reaching its maximum compressive strength, a sudden failure occurs. However, if approx. 1 to 3 vol% steel fibres are added, an increase of ductility in the post-failure area can be achieved [3,4]. High-strength steel fibres are characterized by a tensile strength of 1000–2500 MPa. In general, the fibres have a length of 6–50 mm and a diameter of 0.15–0.5 mm [5]. Even in unloaded concrete, material defects in the form of micro cracks (Figure 1) are present at the interface of aggregate and the cement matrix [6]. The interlocking between fibre and cementitious matrix prevents the development from micro to macro cracks. The fibres allow a transfer of stress between the areas separated by the crack. If the load exceeds a critical value, the micro cracks widen and then evolve to macro cracks. In this phase the fibre should be slowly pulled out to ensure a ductile material behaviour. It is obvious, that the stress transfer depends on the bond properties between the fibre and the surrounding UHPC matrix. On one hand, pullout occurs at a low load if the bond is weak. Thus, the fibres only provide for a small contribution against the propagation of cracks. On the other hand, if the bond is too strong, fibre cracking occurs. Then, no post-cracking behaviour is ensured [7]. Thus, an early rupture of the fibre at low fibre displacements have to be avoided [8].

The stress–slip relationship is used to describe the composite behaviour, i.e., the interaction between fibre and concrete. It can be measured by pullout tests [4,6,9]. The propensity for avoiding a rupture results from the bond properties between the fibres and the concrete matrix [10]. A characteristic value for the fibre behaviour is the utilisation factor (σ_max_/f_y_). An optimum pullout behaviour is given when the utilisation factor of the fibre is up to 80–90% [10,11]. Recent studies revealed [10] that the utilisation factor for straight steel fibres is only 15%. In comparison, fibres with an identical embedding length with end hooks have a utilisation factor of approx. 65%.

### 1.2. Bond Behaviour of Straight and Smooth Fibres

The total bond strength between fibre and cementitious matrix is formed by contributions of adhesive, shear and friction bonds. For straight and smooth fibres, adhesive bonding is the essential mechanism. Upon the adhesive bond reaching its maximum stress, frictional bonding sets in. The characteristics of the frictional bond depend on the bond length, the maximum load and the roughness of the contact Zone (ITZ = interfacial transition zone) between fibre and matrix. The roughness of the fibres also has an indirect influence on the friction bond. The roughness leads to a stronger micro-interlocking and the bond is improved by a larger fibre surface. The crack in the adhesive bond occurs under higher loads and the friction bond is reinforced. Additionally, an increase in the embedding length leads to an increase of the friction bond [10,12]. A shear bond, which is the primary bond in hook-shaped or ribbed steel reinforcements, is not contributing in case of straight fibres [6].

**Figure 2:**
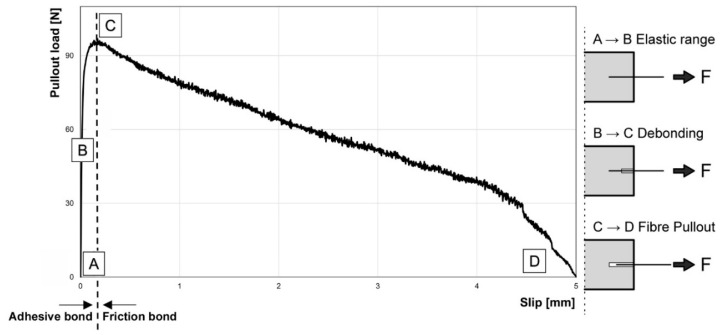
Typical fibre pullout diagram (fibre pullout load versus slip curve) of straight steel fibres (based on [19]).

The adhesive bond results from the chemical and physical interactions between fibres and the concrete matrix [8,13]. This adhesive interaction is mainly influenced by two parts. The first one is chemical adhesion, which is influenced by the degree of hydration, cementitious phases on the fibre surface, attachment of aggregates and air voids in the ITZ [14,15]. The second part of the adhesive bond is mainly ensured by the micro-interlocking (surface quality) of the fibre, as well as the material properties of the components and interface between them (stiffness of the fibre, strength of the cementitious matrix and air voids in the interface area). In addition, on the UHPC matrix side, micro-interlocking can be improved by microfiller or polymer powders such as latex [14] and, on the fibre side, by roughening the surface. The general fibre pullout behaviour of straight steel fibres (brass coated), which are used in numerous investigations [4,16,17], is shown in Figure 2. Upon initiation of the pullout (A), the adhesive bond dominates the behaviour and an elastic fibre elongation takes place. The area B→C is called “Debonding”. A crack is initiated in the contact zone and grows at the interface of the fibre. After complete debonding and reaching the maximum bond force, the friction bond (C→D) dominates the overall behaviour. The pullout force decreases as a function of slip as the embedding length decreases. In contrast to this behaviour, [18] reports a sudden drop of load after reaching the maximum bond force. In this case, steel fibres without coating were used. The different pullout behaviour focusing on coatings will be discussed at the end of this paper.

The bond area between fibre and concrete matrix can be described as a three-phase system consisting of the fibre, surrounding cementitious matrix and the ITZ [7]. This zone is between 30- and 70-µm thick in standard concrete and has a lower strength than the surrounding cementitious matrix. Thus, the micro cracks occur in the ITZ and not directly on the fibre surface. Characteristic for the ITZ is a layer of calcium hydroxide crystals and a slightly higher water content [5,8]. However, the characteristics of the ITZ between the matrix of an UHPC and the fibre surface are still unclear. In any case, the ITZ in case of UHPC is strengthened due to the secondary reaction of silica fume. The Calcium hydroxide (CH) crystals are replaced by C-S-H-phases and the ITZ is reduced in thickness. In [6], SEM images revealed a higher porosity in the direct vicinity of the fibre surface.

The experimental fibre pullout tests in present work provide information about the bond characteristics of UHPC and of different metallic micro fibres. Steel fibres, stainless-steel fibres and shape memory alloy (SMA) fibres of nickel-titanium (NiTi) were used. These were also roughened to increase the bond strength. The conceptualization of the present work is linked to a previous study [20] reporting on a novel functional micro fibre high-performance concrete (FMF-UHPC). By using SMA fibres instead of standard steel fibres, it was expected that it will be possible to provide for a significantly improved and, thus, more efficient UHPC in the future. SMA fibres can “remember” a previous set geometry by the so-called one-way effect. The effect is based on phase transformation of martensite to austenite. The properties of this material pave the way to various fields of application in the building industry [21,22]. The prospects are, for example, continuous prestressing over the whole section or critical areas through SMA fibres in UHPC. The SMA fibres introduced as new reinforcement, could apply such a prestress by thermal activation in the hardened state of the concrete. For this application, the pullout behaviour is essential. Thereby, the stress cannot only be transmitted via anchorage as in conventional prestressing application, but also through the bond of the hardened UHPC and the SMA fibre. The use of SMA fibres in concrete, especially for prestessing, is a part of recent research, in which high potential is seen [23,24].

The results of the single fibre pullout tests shown in [20] revealed that the bond stress of the NiTi-fibres (approx. 1.9 MPa) was about 34% lower than the average bond stress between steel fibres (approx. 2.9 MPa) and the cementitious matrix. However, these results are characterized by strong scatter. In [16], the bonding behaviour of the steel fibres with a thickness of 0.15 mm and an embedding length of 5 mm, which were also used in the present investigations, was studied. Maximum bond stresses of 4.0–5.5 MPa were achieved in the single fibre pullout tests. The maximum bond stresses of fibre series (eight fibres) were approx. 3.0 MPa per fibre. More reliable results were obtained by extracting several fibres from one sample.

The present study deals with the fundamental question to which extent the bonding behaviour of various metallic and straight fibre types and UHPC differs. In order to improve the tensile strength and to ensure resistance during pullout (ductility in the post-cracking area), it is essential to ensure an optimum bonding behaviour. In the literature, steel fibres are often characterised as the same material; however, in many cases the fibre is coated with brass or has other unmentioned structures. This study shows the significant differences in bonding between metallic fibres and UHPC regarding these often unmentioned structures and characteristics. The present work clearly shows that, in case of straight fibres, the bonding is strongly depending on the alloy composition and/or surface modifications. The adhesive as well as frictional bond is considered, without any mechanical contribution by geometric effects. Further, a roughening of the fibre surfaces is shown improve the bond stress. The results of the multiple parallel fibre pullout tests using a CTS device are discussed in combination with results from bending tests in order to be able to conclude on the post-cracking behaviour.

## 2. Materials and Methods

### 2.1. Ultra-High-Performance Concrete (UHPC)

A current, well-known UHPC mixture (UHPC-K18 [25]) was used for the investigations in this study. This mixture is based on the UHPC formulation (M3Q) tested extensively in the SPP 1182 funded by German Research Foundation (DFG) [17], which was adapted to current admixtures. Table 1 shows the compounds and their amounts in relation to a volume of 1 m^3^. The water to binder ratio was 0.21 and the superplasticizer ratio was 1.3 by weight of cement (bwoc). The contents of fibres for the production of 40 × 40 × 160 mm prisms for the determination of the flexural tensile strength were 1.0 and 2.5 vol%.

The concrete has been produced with mixers from Eirich (1 L (EL1) for the samples of the fibre pullout tests and 5 L (EL5) for the flexural tension prisms, Hardheim, Germany). In total, the mixing process lasted 14 min, whereby the raw materials were homogenized for one minute before the addition of water and superplasticizer. The water and superplasticizer mixture were then added during the period of a minute. After this step, the mixing speed was increased. For the sample production, the fibre types were added to the mixture after a mixing break of two minutes by manual trickling. An equal distribution of the fibres was given by this process, so that its effectiveness was not hampered.

**Figure 3:**
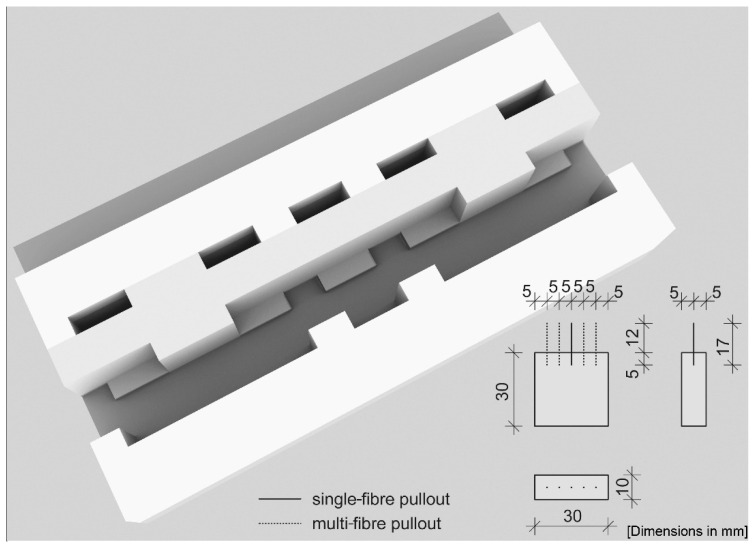
Isometry of the CTS formwork for the production of test samples for single- and multi-fibre pullout tests and the dimensions of the test samples in mm.

For the samples of the fibre pullout test a formwork system has been developed to ensure the exact bond length (Figure 3). The formwork consisted of four individual parts and five inserts, which were used to adjust the fibres. During filling, the formwork was compacted on a vibrating table, 120 s at a frequency of 50 Hz. All samples were removed after 24 h and stored at a temperature of 20 ± 2 °C and a relative humidity of 65 ± 5% before testing.

### 2.2. Fibre Types

**Figure 4:**
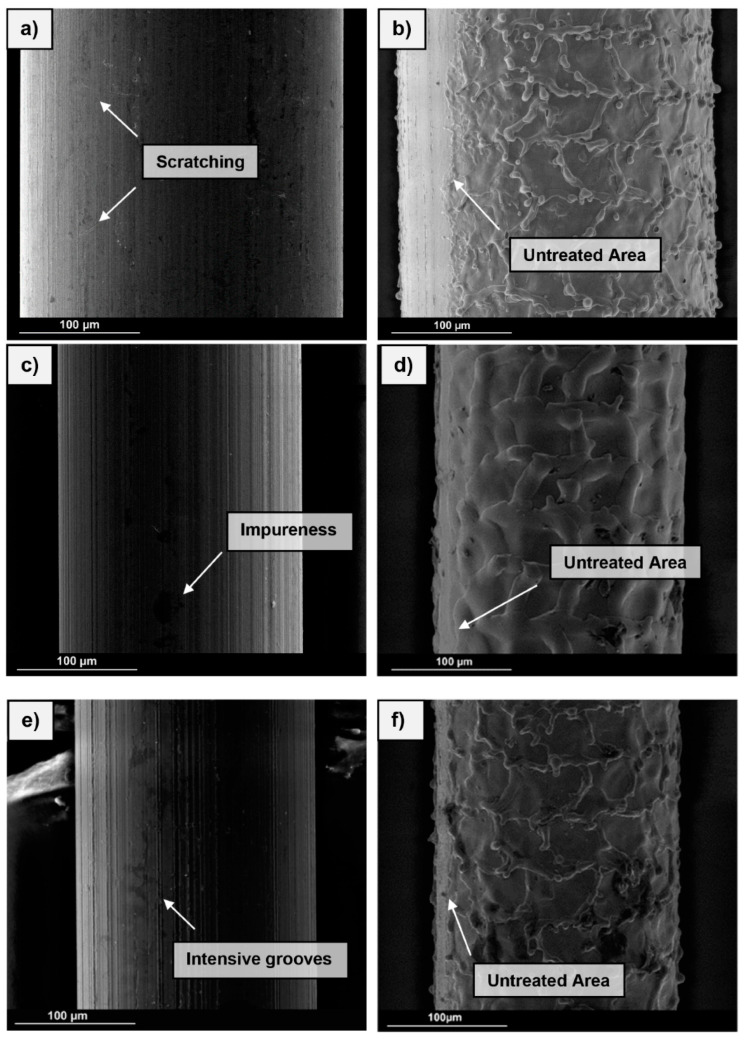
Secondary electron images of the fibre types before the fibre pullout tests. (**a**) Surface of the steel fibres. (**b**) Surface of the steel fibres after roughening. (**c**) Surface of the NiTi-fibres. (**d**) Surface of the NiTi-fibres after roughening. (**e**) Surface of the stainless-steel fibres. (**f**) Surface of the stainless-steel fibres after roughening.

The bond behaviour by fibre pullout tests was studied for three different fibre materials. Brass-coated steel fibres (SF), which are usually used in UHPC to increase the post-breaking behaviour, stainless-steel fibres (SSF) and Nitinol shape memory alloys (NiTi) were used. Figure 4 shows the surfaces of the fibre types recorded by environmental scanning electron microscopy (ESEM). In the following, the properties of the fibre types are described and the surface structures are directly compared.

The brass-coated steel fibres (a) from StraTEC (Stahl-und Fasertechnik, product name: Weidacon FM, Hemer, Germany) consist of high-strength steel and have a tensile strength of approx. 2000 MPa. The SMA fibres made of nickel and titanium (NiTi) (c) have the product name “Alloy M”. The chemical components of the nickel-titanium alloy are almost equiatomic (nickel content 49%). The tensile strength is approx. 1900 MPa [20]. The surfaces of the two fibre types do not show any significant differences (cf. Figure 4). A slight groove in the longitudinal direction can be seen on both fibre types. The steel fibre shows small cracks. The SSF (e) with the designation Acidur 4301 are austenitic chromium-nickel steel fibres being characterised by a high-corrosion resistance. An averaged ultimate tensile strength (UTS, R_m_) R_m_ = 1980 MPa was determined in tensile tests. In comparison to the SF and NiTi, they show a more significant surface topography, i.e., rip like structures parallel to the wire axis.

All used fibres had a length of 17 mm but differed in diameter. The steel fibres had a diameter of 0.25 mm and the two other fibre types had a diameter of 0.20 mm. For calculation of the bond stress, the fibre geometry was considered as well to enable a reasonable comparison of these fibres. Furthermore, in order to improve the bond behaviour of the fibres, surfaces were roughened by a laser (Figure 4b,d,f). For this a Nd: YAG laser system CL100 from CleanLaser GmbH (Herzogenrath, Germany) was used being characterised by a Gaussian laser profile. The laser source is a diode-pumped solid-state laser with an average power of 50 watts and a maximum pulse frequency of 100 kHz. The treated surfaces show a pronounced change of surface topology, i.e., the evolution of characteristic structures, however, being different depending on the fibre type. It should be noted that the fibres were not subjected to this laser procedure over the complete circumference. The fibres were laser treated solely on two opposing surfaces as the fibres were treated individually.

### 2.3. Methods

#### 2.3.1. Fibre Pullout Tests with a CTS-Testing Device

**Figure 5:**
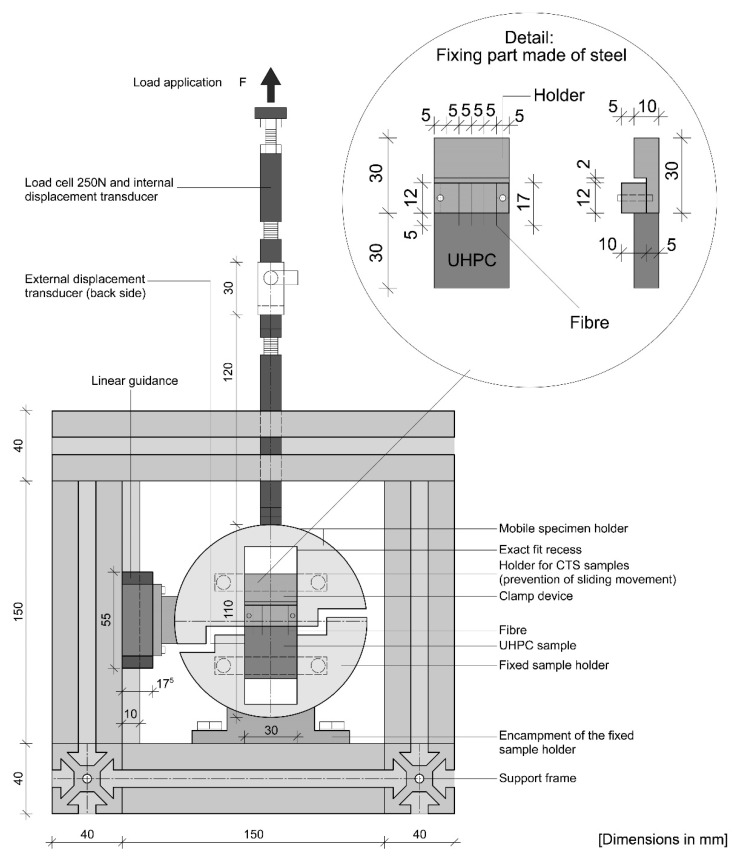
Front-view of the setup for the fibre pullout tests. CTS testing device and fibre fixing holder. All dimensions labelled in mm.

To quantify the bond between the fibres and the cementitious matrix, fibre pullout tests were done using a “Compact-Tension-Shear device” (CTS test). Generally, the CTS device is mostly used to generate tensile, shear and combined tensile-shear loads in case of fracture mechanics tests for metals and plastic bonded structures. Whether a tensile or shear load is applied depends on the angle set for the test fixture. For the fibre pullout tests, the device was installed in tensile position (α = 90°). A detailed drawing of the device and its individual components is shown in Figure 5. The sample dimension was 30 mm × 30 mm × 10 mm, in which the fibres (l = 17 mm) had been embedded over a length of 5 ± 0.5 mm.

The fibre pullout test was done in displacement control with a constant speed of 0.1 mm/s (using a 150 kN Zwick/Roell testing rig). The bond length and pullout speed was chosen based on the results of [20] for better comparability. The fibres were fixed by a steel holder (detail in Figure 5). The CTS sample was installed in the recess of the sample holder and connected to the load line of the testing machine via a threaded bar. The device is made of two sample holders. The sample holder is fixed to the support frame and the upper part can move via a sliding guide.

The pullout force was measured by a 250-N load cell and the traverse displacement was recorded by a transducer. The bond behaviour was determined by pulling the fibre out along the embedding length. In case of fibre failure, no post-cracking behaviour is given. If the desired pullout behaviour occurs, the final pullout stress is lower than the maximum fibre tensile strength.

The maximum bond stress ($\tau_{max}$) is calculated by the following Equation (1):

(1) $$\tau_{max}=\frac{F_{max}}{d_{f}\cdot \pi \cdot l_{e}}$$

*F_max_* is the maximum pullout load, *d_f_* is the fibre diameter and the embedded fibre length *l_e_*. The maximum fibre stress is a measure for the efficiency of the fibre utilization (Equation (2)) [26]:

(2) $$\sigma_{f,max}=\frac{F_{max}}{A_{f}}=\frac{F_{max}}{\pi \cdot \frac{{\left(d_{f}\right)}^{2}}{4}}$$

In order to reflect the actual pullout behaviour, the pullout force is divided by the current compound surface at each slip, i.e., the actual level of displacement. A decreasing area is captured by the following Equation (3), where τ(s) is the bond stress at slip *s* in MPa, and *F*(*s*) is the pullout force at a slip s in N [26]:

(3) $$\tau \;\left(s\right)=\frac{F\;\left(s\right)}{d_{f}\cdot \pi \cdot \left(l_{e}-s\right)}$$

The utilisation factor (*u_f_*) describes the effectiveness of the fibres in percent. It is defined as the ratio of the maximum tensile stress *σ_max_* of the fibre after pulling out to the UTS *f_y_* of the fibre. Here, a utilisation of more than 100% causes fibre cracking. Its value is defined as the ratio of the maximum tensile stress *σ_max_* of the fibre after pulling out to the tensile strength *f_y_* of the fibre (Equation (4)).

(4) $$u_{f}=\frac{\sigma_{f,max}}{f_{y}}\cdot 100$$

#### 2.3.2. Flexural Strength of Fibre-Reinforced UHPC

In order to investigate the influence of the fibre types on the flexural tensile strength, prisms with fibre contents of 1.0 and 2.5 vol% were used. Steel fibres (SF) and stainless-steel fibres (SSF) with different lengths (*l* = 13 mm, *l* = 17 mm) were compared. The mechanical properties, especially the characteristics of the post-cracking behaviour, depend on the fibre length. By using two fibre lengths, a further correlation between the fibre materials is taken into account. It was not possible to produce prisms with NiTi-fibres as reinforcement, as these are not available in large quantities.

The flexural tensile strength of the material was characterised using the same universal testing machine (150kN Zwick/Roell) based on a four-point bending tensile strength test on full prisms of the size 160 mm × 40 mm × 40 mm according to DIN EN 12390-5 [27]. The test was done at a constant crosshead displacement speed of 0.01 mm/s. To reduce the scatter of values, a homogeneous fibre distribution and orientation was ensured by filling the prisms in three layers using a filling chute. In addition, the prisms were tested in filling direction. Thus, the failure of the sample mainly occurs downface of the prisms, finally reducing the error of measurement. It is assumed that the side faces of the prisms are subject to higher variations due to fibre orientation and distribution.

For the results of the post-cracking behaviour of the fibre variations, the average flexural strength at the point of deflection $\delta_{L1}=0.5\;\mathrm{mm}$ for the characteristic post-cracking flexural strength of the service load range $(f_{cflk,\;L1}^{f}$) and $\delta_{L2}=3.5\;\mathrm{mm}$ in the fracture range ($f_{cflk,L2}^{f}$) as well as from the maximum load were compared. These ranges are defined in accordance with the DAfStb-guideline “Stahlfaserbeton” [28]. The average tensile strength at the characteristic points [MPa] are determined using the following Equations (5) and (6) [28]:

(5) $$f_{cflm,L1}^{f}=\frac{1}{n}\overset{n}{\underset{i=1}{\sum }}\frac{M_{0.5i}}{W_{0.5i}}=\frac{1}{n}\overset{n}{\underset{i=1}{\sum }}\frac{\frac{F_{0.5i}}{2}\cdot \frac{l}{3}}{\frac{b_{i}\cdot h_{i}^{2}}{6}}=\frac{1}{n}\overset{n}{\underset{i=1}{\sum }}\frac{F_{0.5i}\cdot l}{b_{i}\cdot h_{i}^{2}}$$

(6) $$f_{cflm,L2}^{f}=\frac{1}{n}\overset{n}{\underset{i=1}{\sum }}\frac{F_{3.5i}\cdot l}{b_{i}\cdot h_{i}^{2}}$$

where *F* is the applied load [N], *l* is the support distance [mm], *b* is the section width [mm], *h* is the section height [mm], *M* is the moment [Nmm], *W* is the section modulus [mm^3^] and the index *i* is the sample number.

Furthermore, a classification can be made on the basis of these areas and a performance factor can be determined. For this classification the characteristic values of the cracking flexural strength are defined. These are determined as a function of v as follows Equations (7) and (8):

(7) $$\begin{array}{cc} if\;v\leq 0,25: & f_{cflk,Li}^{f}=0,51\cdot f_{cflm,Li}^{f} \end{array}$$

(8) $$\begin{array}{cc} if\;v>0,25: & f_{cflk,Li}^{f}=f_{cflm,Li}^{f}\cdot \left(1-t\cdot v\right) \end{array}$$

where v is the coefficient of variation and t the threshold for t distribution (5%—fractile).

#### 2.3.3. Microstructural Investigations—Scanning Electron Microscopy (SEM)

The fibre types were studied by using an ESEM. The surface morphologies and textures, respectively, of the fibres were considered before and after fibre pullout in order to establish relationships between the micro-interlocking and the bond of cementitious phases. For comparison, the boundary and composite areas of the fibres were characterised. The ESEM used was a Quanta FEG 250 from FEI (Field Electron and Ion Company). The pictures were taken in secondary electron (SE) mode (Low-vacuum, voltage 15 kV, working distance 10 mm).

## 3. Results

### 3.1. Fibre Pullout Tests—Influence of the Fibre Type on the Bond between Fibre and Cementitious Matrix

**Figure 6:**
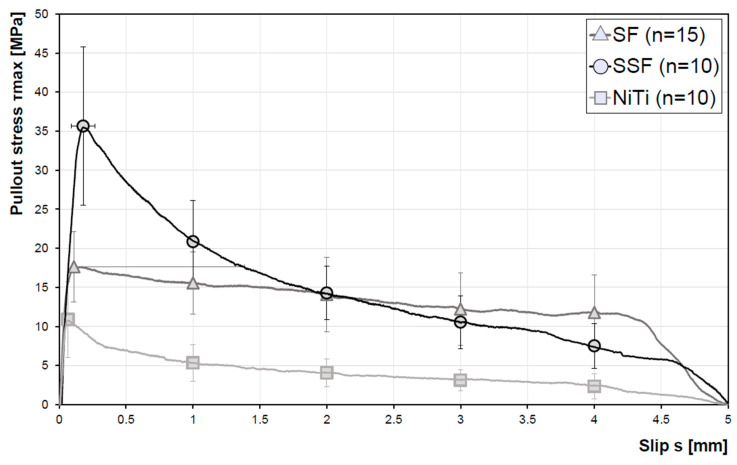
Average fibre pullout stress versus slip curves for the different fibre types without surface treatment.

The characteristic stress/slip relationship of several samples of the different micro fibre types without surface treatment is highlighted in Figure 6. The number of samples examined was 15 for the brass-coated steel fibres (SF) and 10 for the stainless-steel fibres (SSF) and the NiTi fibres, respectively. The bond stresses are based on the extraction of 5 fibres for each fibre material. The results show that the micro fibres differ significantly in their bond behaviour. The stainless-steel fibres (SSF) show the highest maximum bond stress of approx. 36 MPa. However, a fast decrease of the bond stress after reaching the maximum stress is noticeable. The bond stress of the SSF decreases rapidly over the first 2 mm, by about 2.5 times with respect to the maximum value. In contrast, the NiTi fibres represent the weakest bond with the concrete matrix, the maximum pullout stress is about 11 MPa. The steel fibres have a higher variability in the range of the maximum bond stress as well as in the slip at maximum bond stress. In the friction bond regime higher pullout forces were found in comparison to the SF- and SSF-fibres. At a fibre pullout of 2 mm, the stress is in the range of the SSF.

The pullout curve shown in the introduction section clearly is met by these results. In the first part, which is defined as area A→B in Figure 2, the pullout behaviour shows an elastic, linear behaviour and the fibre deforms. At the end of the elastic pullout phase, the separating phase begins. Subsequently, the physical adhesion and static friction are reduced. With progressing pullout a reduction of the pullout force is visible and the micro fibre is finally pulled out. The SSF show the highest utilisation factor with 36.0%. The SF have a factor of 14.1% and the NiTi-fibres of 12.1% (Table 2).

### 3.2. Fibre Pullout Tests—Influence of Surface Topography on the Bond between Fibre and Cementitious Matrix

**Figure 7:**
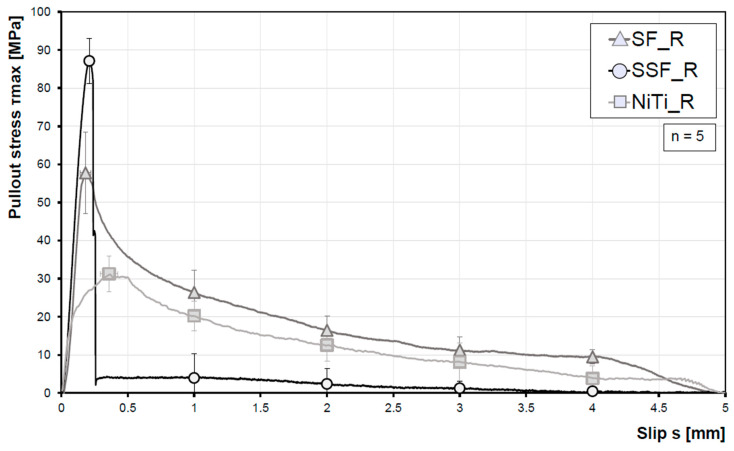
Average fibre pullout stress versus slip curves for the different fibre types.

The characteristic stress/slip relationship for the roughened fibre types is shown in Figure 7. For each fibre type, five samples were examined. The SSF with a maximum value of ~87 MPa also show the highest maximum pullout stress, which is about 53% higher as compared to the SF (~57 MPa). The lowest values for pullout stress (~31 MPa) were found for the NiTi-fibres. The strong decrease in the pullout stress at low fibre displacement of the SSF resulted from failure of fibres. Still, a low bond stress is seen induced by the friction bond, because only four of the five fibres cracked. In the case of the SF and NiTi-fibres, no fibre rupture occurred and a complete pullout was achieved. Furthermore, it is noticeable that the maximum pullout stress of the NiTi-fibres occurs at a later slip (~0.4 mm) compared to the other two fibre types. Compared to the smooth fibres, the utilization factor of all roughened fibres is on a higher level (Table 3). The SSFs have the highest utilisation factor at 88%. After roughening of the fibre surface, NiTi-fibres are in the range of SSF without roughening.

**Figure 8:**
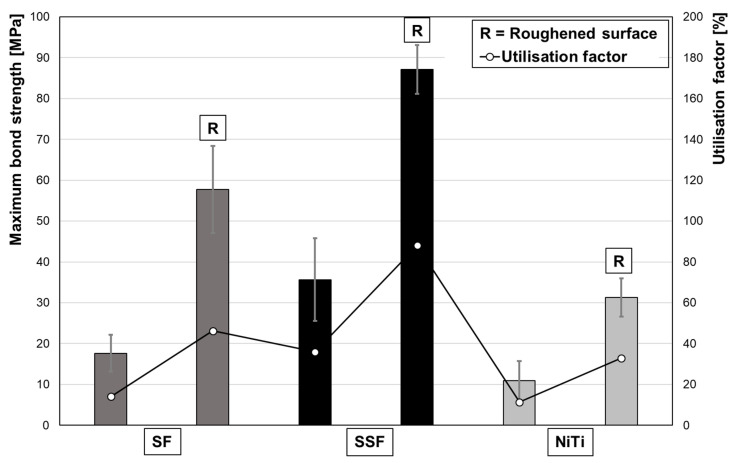
Influence of surface topography on the bond between fibre and concrete matrix. Maximum bond stress and utilisation factors of the untreated and roughened surfaces of the different fibre types.

In a direct comparison, the modified surface topography results in significantly higher pullout stresses (Figure 8). Due to the roughened surface, the maximum pullout stresses for SF, SSF and NiTi are ~3.28, ~2.44 and ~2.87 times higher, respectively.

### 3.3. Microstructural Analysis

**Figure 9:**
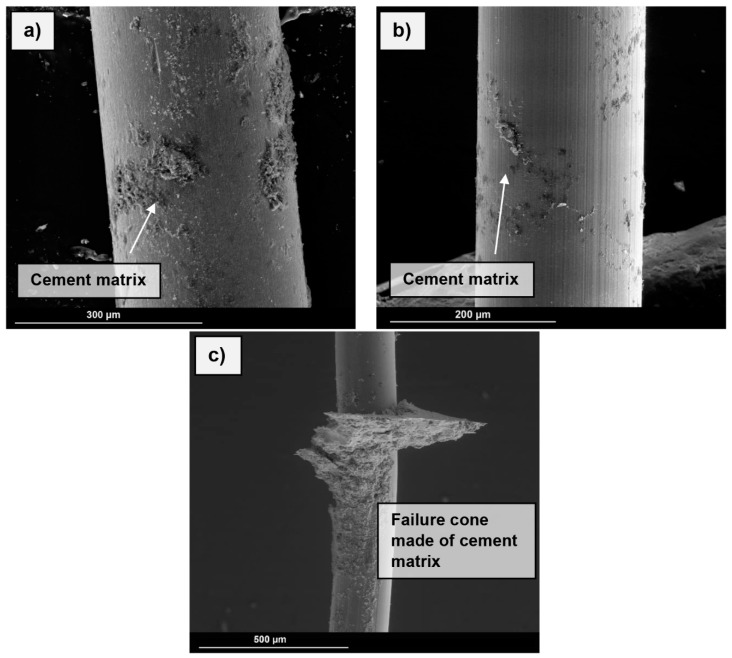
Secondary electron images of the embedded parts of the pullout fibres; (**a**) Boundary between the embedded and blank part of a SF. (**b**) Boundary between the embedded and blank part of a SMA fibre. (**c**) Boundary between the embedded and blank part of a SSF. Image in low-vacuum mode, voltage 15 kV, working distance 10 mm.

The secondary electron (SE) images shown in Figure 9 are used to characterise the bond of the cementitious matrix, such as C-S-H-phases present in the boundary zone (area between embedded and blank fibre). In the case of steel fibres (Figure 9a), plane matrix components are visible in the boundary zone. In addition, there are isolated areas with attached cementitious phases over the full length of the fibre. In contrast, only punctual attachment can be seen in the boundary zone of the NiTi fibres (Figure 9b). When considered over the entire interconnected area, no agglomerates were visible below the boundary area. The highest maximum bond strength was provided by the SSF. Figure 9c shows large attachments of cementitious matrix in the boundary zone and a failure cone has formed.

**Figure 10:**
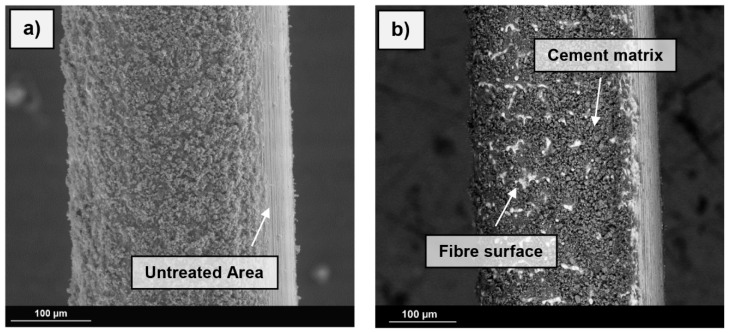
(**a**) Roughened steel fibre after pullout (SE-Image). (**b**) Roughened steel fibre after pullout (BSE-image). Low-vacuum mode, voltage 15 kV, working distance 10 mm.

The SE- (Figure 10a) and BSE-images (Figure 10b) show the effect of the roughened surface based on the example of steel fibres. The untreated areas are clearly visible, as there are only minimal attachments in this area. The remaining fibre surface is completely covered with cementitious parts. The backscattered electron image shows the material contrast, thus, areas including parts with higher density appear brighter. The image clearly shows the high bond of the parts of the cement matrix on the fibre surface. The spaces between the high points of the fibre surface, i.e., the surface topography maxima (ct. Figure 4), are covered with the concrete matrix. The horizontal ripples caused by the roughening process can be seen through the cement matrix.

### 3.4. Flexural Strength—Influence of the Fibre Type

**Figure 11:**
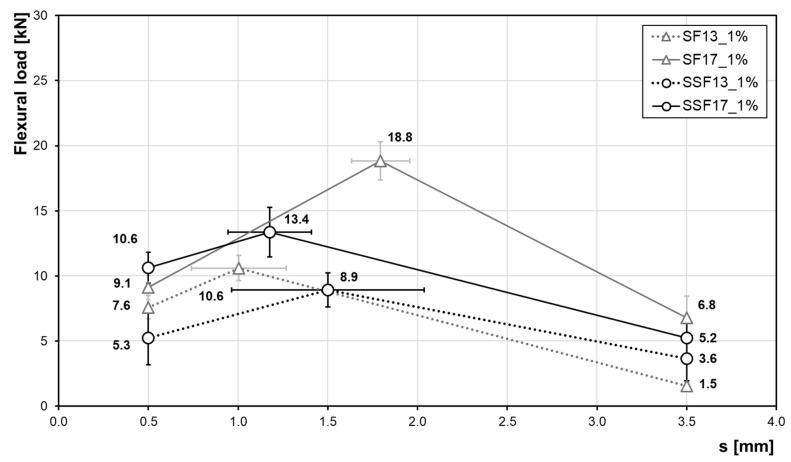
Influence of fibre material and length (at a constant volume fraction of 1.0 vol%) on the flexural strength. Data shown highlight the flexural load at a distance of 0.5 mm, 3.5 mm and the maximum load including standard deviations.

The force-displacement diagram (Figure 11) shows the results of the test samples with steel and stainless-steel fibres for a fibre content of 1.0 vol%. Due to scattering, the determination of a mean curve from the curves of the individual fibre types is difficult. Thus, results are represented by three points introduced in the DAfStb-guidline [28], which reflect the characteristic ranges and considering standard deviations. Outlier samples, i.e., strongly scattering measured values, were taken out. In total, nine prisms were tested from each test series. Eventually, the mean course is represented by six samples in each case.

For all investigated fibre types and lengths, the force increases after the first crack. The general requirement, i.e., to delay a crack propagation and ensure post-cracking behaviour, is therefore achieved in case of all fibre reinforcements considered. In a direct comparison of two different fibre types, i.e., SSF and SF with a fibre length of 13 mm, SF13 are characterised by a maximum load that is ~19% higher than in case of SSF13. However, a high standard deviation has to be taken into account here. For the SF13 fibres the force increases by ~40% from the service load range (point of deflection $\delta_{L1}=0.5\;\mathrm{mm}$) to the maximum load. Afterwards, the force decreases steeply up to a deformation of 3.5 mm (fracture load range). The maximum forces of the SSF13 samples are reached at larger deformations at about 1.5 mm. Compared to the SF fibres, the SSF fibres provide for a higher working capacity in the post-cracking area by reaching the maximum load only at 1.5 mm. This behaviour could not be observed in the 17-mm-long fibres. For the SF17, the maximum load is achieved at a deformation of 1.8 mm, while the maximum load of SSF is already achieved at 1.2 mm. The maximum force level seen in case of the SSF samples is ~42% lower than for the prisms with SF fibres. In general, the longer fibres ensure a more post-cracking behaviour, which is shown by a higher flexural strength for large deformations.

Table 4 lists all values determined for a constant fibre content of 1.0 vol%. Summarised are the average values of the maximum force and the reciprocal flexural strength. Furthermore, the performance factor serves for a direct comparison of the average forces in the service range and the fracture range.

**Figure 12:**
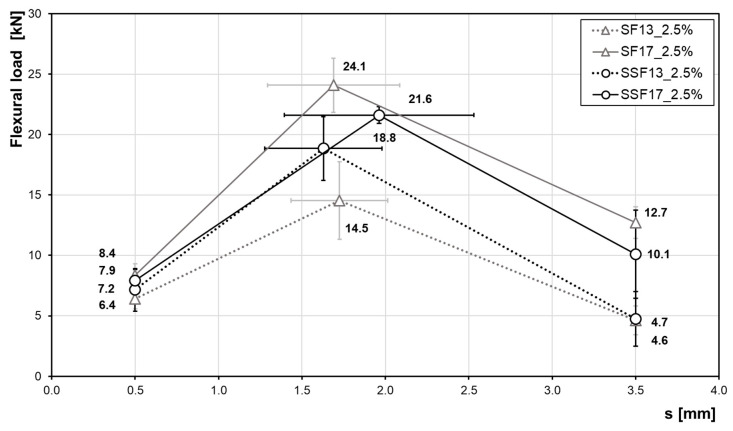
Influence of fibre material and length (at a constant volume fraction of 2.5 vol%) on the flexural strength. Data shown highlight the flexural load at a distance of 0.5 mm, 3.5 mm and the maximum load including standard deviations.

An increase of the fibre content to 2.5 vol% (Figure 12 and Table 5) leads to an even more increased flexural strength. Compared to a fibre content of 1.0 vol%, the maximum forces and the performance at high deformations of 3.5 mm have clearly increased. Thus, the samples exhibit an improved pseudo-ductile post-cracking behaviour as more fibres are activated in the crack area. When comparing the fibre materials both with a length of 13 mm, the gradient up to the maximum load of samples with SSF is higher as compared to SF fibres. Compared to each other, the maximum load of SSF is ~30% higher. In case of fibres with a length of 17 mm, the samples with steel fibres cause a higher maximum load compared to the SSF. For smaller deformations, there is no significant difference between the fibre types.

Considering the two longer (17-mm) fibre types in terms of their mechanical properties, the flexural strength is significantly higher than those of the shorter fibres. In this case, especially the performance in the fracture range (s = 3.5 mm) is significantly improved as the flexural load is more than two times higher, i.e., a ductile behaviour over a wider range is ensured.

## 4. Discussion

The focus of this study was the analysis and quantification of the bond behaviour of metallic micro fibres and a cementitious matrix. The simultaneous pullout of five fibres using a CTS device proved to be a suitable method for this purpose as the results are in good agreement with the typical bond–stress curve in the literature [29]. Compared to single-fibre tests [20], the results were found to be more precise and characterised by less scatter, respectively.

The results of the untreated fibres showed significant differences in bond behaviour for each fibre type. The brass-coated steel fibres (SF) had a maximum pullout stress of approx. 18 MPa, which corresponds to 3.6 MPa per fibre. This result is in line with data published in the literature [16]. Furthermore, the steel fibres showed a relatively high curve level over the frictional bond (Figure 6). However, compared to the other fibre types, these fibres vary more significantly in terms of the range of the maximum bond stress. For some of the examined samples, the maximum was only reached in a relatively late slip step (standard deviation over a slip range of approx. 1.3 mm). The pullout stress steadily decreases throughout the friction bond regime. A factor that clearly describes this behaviour is the pullout work (W_p_). This value is defined as the area under the pullout load versus slip curve [19,30]. In the present study, this is represented by the integral over the entire embedding length of 5 mm. The SF (brass coating) is characterised by a W_p_ = 246⋅10^−3^ J and, thus, a higher work level as compared to the SSF with W_p_ = 219⋅10^−3^ J and NiTi-fibres with only W_p_ = 61⋅10^−3^ J. This is somehow contradictory to the literature [18], where the maximum bond stress of SF without brass coating mostly is reached at a relatively low slip dropping abruptly afterwards. The different bond behaviour of the SF is explained by the brass coating, whereupon a higher working behaviour is achieved over the friction bond. The commercially available fibres with brass coating [16,31] used here show a promising friction bond over the pullout. However, further analysis of the effect of the brass coating is beyond the scope of present work. The bond behaviour of the SSF, which is without any coating, fits well with the results from the literature [18].

Compared to the steel fibres, the stainless-steel fibres show a bond stress that is about twice as high. One factor for this increase is thought to be the slight scoring in case of the fibre surface of the SSF (Figure 4e). Thus, compared to the SF and NiTi-fibres, this type of fibre could promote a higher number of micro-interlocking sites. An argument in favour of this assumption is a strong curve drop after reaching the maximum bond stress. After a slip of 2 mm, the bond stress is lower than that of the SF. Due to the surface topography, an increased surface area is available, contributing to the adhesive bond, however, playing a minor role through the friction bond.

The NiTi showed the weakest bond to the UHPC matrix, even though the fibres showed only slight differences in surface roughness compared to the SF. Accordingly, the interlock of cementitious phases between the micro fibre material and concrete matrix has a significant influence on the bonding behaviour. The bond strength varies depending on the alloy composition and eventually the surface characteristics imposed thereby. This can also be seen in the SEM images (Figure 11), revealing only slight attachments of cementitious matrix on the fibre surface. Other factors can be related to the mechanical properties of the fibre. A higher stiffness improves the bond properties [32]. In [32], the stiffness of NiTi fibres is increased by cold drawing, which leads to an improved pullout strength. The influencing factor here is the contact pressure, which results from the shrinkage of the cementitious matrix in the fibre–matrix interface. In contrast, a lower stiffness would have a negative effect on the adhesive bond.

When fibres are used as a reinforcement or prestressing element, the bond quality is of utmost importance. With a utilization factor of ~11% in case of non-surface-treated NiTi, the efficiency is well below the optimum range [10]. By roughening the fibre surfaces, it was possible to increase the maximum bond stress. This effect is based on the significant promotion of micro-interlocking. Furthermore, the changed surface topography leads to a larger bonding surface, which increases the strength between the fibre and the UHPC-matrix. Additionally, the formation of grooves orthogonal to the pullout direction likely has a positive effect, since the roughened surface following the laser treatment facilitates the anchorage of the cement matrix, and thus, significantly increases the bond strength. The SEM images of the laser treated fibre surfaces of the SF and SSF showed a similar structure. However, the increase in pullout stress due to surface treatment is different for both fibre types. The largest increase was found for the SF with ~228%. The increase for the SFF was only ~144%, but this can be explained by the fact that the potential of the fibres was exhausted and a fibre tearing occurred in the tests. Induced by the roughening treatment, the bonding behaviour of the SFF was strongly affected. With a utilisation factor of 88%, the value upon treatment is within the optimum range specified in the literature [10,11]. However, a tearing of the fibres occurs. One reason for this could be an uneven pulling of the fibres, resulting in an irregular stress distribution on the five fibres. Furthermore, it has to be taken into account that the laser surface treatment changes the microstructures of the envisaged metallic alloys due to a concomitant thermal treatment. The laser surface treatment induces high local energy only for a very short time eventually promoting rapid increase in temperatures very locally. Thus, in the thin fibres considered in present work not only changes of surface appearance, as clearly revealed by ESEM characterisation (Figure 4), have to be taken into account. Furthermore, significant evolution of microstructure in the whole wire cross-section is expected to be enabled. Eventually, the mechanical performance of the treated wires will be affected by the surface and the bulk microstructural characteristics, e.g., martensitic transformation and appearance of secondary phases, surface topography, residual stress, thickness of the affected layer and other factors in a highly combined fashion. The texturing of surfaces by laser treatment has been in focus of research for decades and, thus, ample examples can be found in the literature detailing complexity of these interrelations [33,34,35,36]. As the focus of present work was on the evaluation of the performance of the fibre-reinforced UHPC compound behaviour, an in-depth analysis of the mechanical behaviour of single fibres is clearly beyond the scope of present work. Results will be published in follow-up studies. Most importantly in case of present findings, it has to be noted that the positive effect of the laser surface treatment was less pronounced for NiTi. The increase was ~41% below that for the SF. This behaviour can already be expected upon closer evaluation of the ESEM images of the fibres after the laser process (Figure 4). The surface structure of the NiTi differs from the other fibre types qualitatively. The grooves seen are less deep and the structures in general are smoother. With a utilization factor of 46.2 for the SF and 34.8 for NiTi, the bond behaviour is expected to be further enhanced by optimized profiling. In this respect, the different fibre types will be alternatively treated in further investigations in order to address the influence of the surface condition in more depth.

A direct correlation of pullout tests of the fibres with the flexural strength of prisms seems unreasonable based on the results obtained in present work. At a fibre length of 13 mm and a fibre content of 1.0 vol%, the values determined for the SF and SSF prisms do not differ significantly. Still, the ~19% higher flexural strength of the SF prism contrasts with the superior bond behaviour of the SSF in the fibre pullout tests. At a fibre content of 2.5 vol%, the results reveal a superior behaviour on the side of SSF. The maximum flexural strength is ~30% higher than in case of SF. Concerning the different length of fibres, for the 17-mm-long fibres the flexural strength of SF is significantly higher with 1.0 vol% and still slightly higher with 2.5 vol% as compared to SSF. Due to many other influencing parameters, such as fibre inclination, slight changes in the geometry of the fibres, positioning and the abundance of fibres in the fracture zone, the influence of aggregates—which, up to now, are a direct comparison of results obtained by pullout tests with values obtained by flexural strength tests—should be evaluated carefully [26,37]. In [26], the flexural strength of UHPC beams with hook-shaped fibres and straight steel fibres, both with a length of 30 mm, was determined. In that study, the results of the pullout tests showed an improved bond performance, however, on the contrary, a decrease in the flexural strength for the hooked fibres. However, no cracking of the fibres was observed. As well as in the present study, a clear rationale could not be provided in that case. Therefore, in future investigations, one of the major challenges will be to minimize the scatter of results to improve statistics. Regarding the use of NiTi, it is important that in the next steps a large quantity of fibres is available to produce prisms for flexural strength testing. Furthermore, additional functional fibres will be considered. In terms of large-quantity fibre applications, cost-effective alternative SMAs, e.g., based on iron as primary element [38,39,40,41,42], seem to be more promising than the application of NiTi. However, studies reporting on compounds made from these alloys have not been reported so far. In any case, the impact of the surface treatment on the functional response has to be considered carefully as SMAs are very sensitive to any change of microstructure and surface condition.

## 5. Conclusions

The effect of different fibres and a roughening of the fibre surface on the pullout behaviour from the UHPC matrix was investigated and discussed. In order to improve the tensile behaviour and to ensure a resistance pullout (ductility in post-cracking behaviour), it is essential to ensure an optimal bond behaviour. For the investigation, multi-fibre pullout tests were carried out with a CTS device. Furthermore, the flexural behaviour of prisms with different fibre types and contents were investigated. Based on the results, the following conclusions can be drawn:

(1) In general, the fibre pullout tests have shown that the interlock of cementitious phases with the fibre surface of smooth fibres is an essential factor contributing to the strength of the bond. The SFF show a significantly higher bond strength as compared to the SF. Compared to the SF, the NiTi-fibres have the lowest bond strength. Further factors to be considered at this point are the mechanical properties (stiffness) of the fibres and topography, surface structures and texture of the fibres.
(2) The CTS device can be rated as reliable due to its mechanical stability and a precise fitting that ensures a centric pullout. Results can be achieved more robustly by pulling out multiple fibres simultaneously.
(3) A roughening of the fibre surface significantly increases the strength of the adhesive bond. The resulting ITZ plays a decisive role, in conjunction with the surface structure. Micro interlocking improves the bond behaviour in the contact area between the fibre and the UHPC matrix. A roughening has a different effect for each type of fibre. The most significant difference compared to the fibres with a smooth surface was found for the SF. Furthermore, the roughening process has improved the bond between SFF and UHPC to such an extent that the fibres failed.
(4) The difference of the flexural strength in case of prisms with SF and SFF is not that obvious as compared to results obtained by fibre pullout testing. For a fibre content of 1.0 vol% and a fibre length of 17 mm, the SF have a higher flexural strength compared to the SFF with equal length. Thus, a correlation between fibre pullout and flexural strength cannot be established successfully, most likely due to the complexly combined effect of multiple influencing parameters.
(5) The maximum flexural strength found for the 17 mm fibres is higher than in case of fibres with a length of 13 mm. Of great importance is the fact that the performance under fracture conditions is significantly improved. Furthermore, the maximum flexural strength occurs during later stages of deformation. Additionally, by increasing the fibre content, the strength increases. However, both factors, i.e., fibre content and fibre length, are associated with a decrease in workability of the fresh concrete.

## Figures and Tables

**Table 1:** Compounds of the M3Q mixture and their amounts in kg/m^3^ and wt % related to dry materials.

Compounds	kg/m^3^	wt %
Cement: CEM I 52.5 R HS/NA	801	34.0
Silica fume: Sika Silicoll P	170	7.2
Quartz sand: G32 0.125 mm/0.5 mm	970	41.2
Quartz powder: Millisil W12	199	8.5
Superplasticizer: Sika Viskocrete 2810	24	1.3 bwoc
Water	190	8.1
w/b-ratio *	0.21	

* w/b: water/binder ratio.

**Table 2:** Results of the fibre pullout tests for the different fibre types.

Designation	*F_max_*	*F_max_/fibre*	*f_y_*	*d_f_*	*sF_max_*	*τ_max_*	σ *_f,max_/fibre*	*u_f_*
Unity	N	N	MPa	mm	mm	MPa	MPa	%
SF	69.2	13.8	2000	0.25	0.11	17.6	282	14.1
SSF	112	22.4	1980	0.20	0.18	35.7	713	36.0
NiTi	34.2	6.8	1900	0.20	0.07	10.9	218	11.4

*F_max_*: maximum pullout load, $F_{max}/fibre$: maximum pullout load per fibre, *f_y_*: tensile strength of the fibre, *sF_max_*: fibre pullout at maximum pullout load, *d_f_*: fibre diameter, $\tau_{max}$: maximum bond stress, $\delta_{f,max}$: Maximum reached fibre tensile stress, *u_f_*: Capacity utilization of the fibre.

**Table 3:** Results of the fibre pullout tests for the different fibre types following laser surface treatment.

Designation	*F_max_*	*F_max_/fibre*	*f_y_*	*d_f_*	*sF_max_*	τ *_max_*	σ *_f,max_/fibre*	$u_{f}$
Unity	N	N	MPa	mm	mm	MPa	MPa	%
SF_R	226.8	45.4	2000	0.25	0.18	57.7	924	46.2
SSF_R	273.6	54.7	1980	0.20	0.21	87.1	1742	88.0
NiTi_R	98.3	19.7	1900	0.20	0.36	31.3	626	32.9

*F_max_*: maximum pullout load, $F_{max}/fibre$: maximum pullout load per fibre, *f_y_*: tensile strength of the fibre, *sF_max_*: fibre pullout at maximum pullout load, *d_f_*: fibre diameter, $\tau_{max}$: maximum bond stress, $\delta_{f,max}$: Maximum reached fibre tensile stress, *u_f_*: Capacity utilization of the fibre.

**Table 4:**
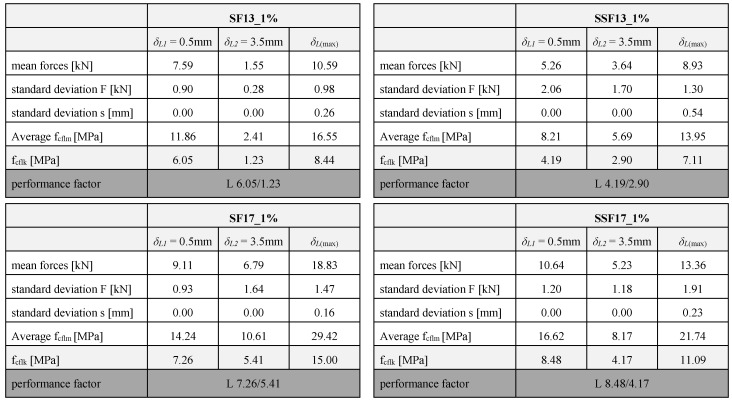
Results of the flexural strength of the fibre material and length (constant fibre volumes of 1.0 vol%) and classification into performance classes.

**Table 5:**
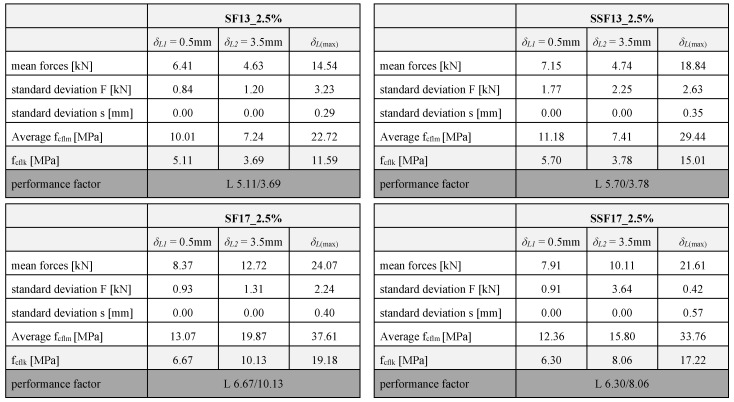
Results of the flexural strength of the fibre material and length (constant fibre volumes of 2.5 vol%) and classification into performance classes.

## References

[1] Fehling E., Schmidt M., Teichmann T., Middendorf B. (2005). Entwicklung, Dauerhaftigkeit und Berechnung Ultra—Hochfester Betone (UHPC).

[2] Leutbecher T., Fehling E. (2012). Tensile Behavior of Ultra-High-Performance Concrete Reinforced with Reinforcing Bars and Fibers: Minimizing Fiber Content. ACI Struct. J..

[3] Wu Z., Shi C., He W., Wu L. (2016). Effects of steel fiber content and shape on mechanical properties of ultra high performance concrete. Constr. Build. Mater..

[4] Fröhlich S., Schmidt M. (2014). Testen Von Ultra—Hochfestem Beton. Universität Kassel.

[5] Wietek B. (2010). Stahlfaserbeton. Grundlagen und Praxisanwendungen.

[6] Leutbecher T. (2007). Rissbildung und Zugtragverhalten von Stabstahl und Fasern bewehrtem Ultrahochfesten Beton (UHPC). Ph.D. Thesis.

[7] Abdallah S., Fan M., Rees D.W.A. (2018). Bonding Mechanisms and Strength of Steel Fiber-Reinforced Cementitious Composites: Overview. J. Mater.Civ. Eng..

[8] Holschemacher K., Klug Y., Dehn F., Wörner J.-D., Bergmeister K., Wörner J.-D. (2006). Faserbeton, Betonkalender 2006, Teil 1. Turmbauwerke, Industriebauten.

[9] König G., Holschemacher K., Dehn F. (2002). Faserbeton—Innovation im Bauwesen, Beiträge aus Praxis und Wissenschaft.

[10] Breitenbücher R., Song F. (2014). Experimentelle Untersuchungen zum Auszugsverhalten von Stahlfasern in höherfesten Betonen. Beton Stahlbetonbau.

[11] Markovic I. (2006). High-Performance Hybrid-Fibre Concrete Development and Utilisation.

[12] Kim B.-J., Yi C., Ahn Y.-R. (2017). Effect of embedment length on pullout behavior of amorphous steel fiber in Portland cement composites. Constr. Build. Mater..

[13] Naaman A.E., Namur G., Alwan J., Najm H. (1991). Fiber Pullout and Bond Slip. I: Analytical Study. J. Struct. Eng..

[14] Naaman A.E. (2000). Fasern mit verbesserter Haftung. Beton Stahlbetonbau.

[15] Ostrowski K., Sadowski Ł., Stefaniuk D., Wałach D., Gawenda T., Oleksik K., Usydus I. (2018). The Effect of the Morphology of Coarse Aggregate on the Properties of Self-Compacting High-Performance Fibre-Reinforced Concrete. Materials.

[16] Stürwald S. (2011). Versuche Zum Biegetragverhalten von UHPC Mit Kombinierter Bewehrung.

[17] Schmidt M. (2013). Sustainable building with ultra-high performance concrete (UHPC)—Coordinated research program in Germany. Res. Appl. Struct. Eng. Mech. Comput..

[18] Cunha V.M.C.F., Barros J., Sena-Cruz J. (2008). Bond-Slip Mechanisms of Hooked-End Steel Fibers in Self-Compacting Concrete. Mater. Sci. Forum.

[19] Qi J., Wu Z., Ma Z.J., Wang J. (2018). Pullout behavoir of straight and hooked- end steel fibers in UHPC matrix with various embedded angles. Constr. Build. Mater..

[20] Schleiting M., Wetzel A., Krooß P., Thiemicke J., Niendorf T., Middendorf B., Fehling E. (2020). Functional microfibre reinforced ultra-high performance concrete (FMF-UHPC). Cem. Concr. Res..

[21] Cladera A., Weber B., Leinenbach C., Czaderski C., Shahverdi M., Motavalli M. (2014). Iron-based shape memory alloys for civil engineering structures: An overview. Constr. Build. Mater..

[22] Janke L. (2005). Applications of shape memory alloys in civil engineering structures—Overview, limits and new ideas. Mater. Struct..

[23] Czaderski C., Shahverdi M., Brönnimann R., Leinenbach C., Motavalli M. (2014). Feasibility of iron-based shape memory alloy strips for prestressed strengthening of concrete structures. Constr. Build. Mater..

[24] Moser K. (2005). Feasibility of concrete prestressed by shape memory alloy short fibers. Mater. Struct..

[25] Wetzel A., Piotrowski S., Reinhardt L., Middendorf B. (2018). Quarzmehlfreier UHPC mit Kalkstein-oder Basaltmehl. Beton Stahlbetonbau.

[26] Yoo D.-Y., Park J.-J., Kim S.-W. (2017). Fiber pullout behavior of HPFRCC: Effects of matrix strength and fiber type. Compos. Struct..

[27] DIN EN 12390-5 (2017). Testing Hardened Concrete—Part 5: Flexural Strength of Test Specimens.

[28] DAfStb-Richtlinie: Stahlfaserbeton (2008). Ergänzung zu DIN 1045.

[29] Lanwer J., Oettel V., Empelmann M., Höper S., Kowalsky U., Dinkler D. (2019). Bond behavior of micro steel fibers embedded in ultra-high performance concrete subjected to monotonic and cyclic loading. Struct. Concr..

[30] Wille K., Naaman A.E. (2013). Effect of ultra-high-performance concrete on pullout behavior of high-strength brass-coated straight steel fibers. ACI Mater. J..

[31] Fehling E., Leutbecher T., Thiemicke J. (2014). Zum Zugtragverhalten von UHPC mit kombinierter Bewehrung.

[32] Kim D.-J., Kim H.A., Chung Y.-S., Choi E. (2014). Pullout resistance of straight NiTi shape memory alloy fibers in cement mortar after cold drawing and heat treatment. Compos. Part B Eng..

[33] Etsion I. (2005). State of the Art in Laser Surface Texturing. J. Tribol..

[34] Bonse J., Hohm S., Kirner S.V., Rosenfeld A., Kruger J. (2016). Laser-Induced Periodic Surface Structures—A Scientific Evergreen. IEEE J. Sel. Top. Quantum Electron..

[35] Greiner C., Schäfer M. (2015). Bio-inspired scale-like surface textures and their tribological properties. Bioinspiration Biomim..

[36] Taube A., Kurtovic A., Niendorf T., Mertens T., Zinn C., Schaper M., Maier H.J. (2016). Influence of laser-assisted surface pre-treatments on the high-cycle fatigue behaviour of TiAl6VInt. J. Fatigue.

[37] Cattaneo S., Muciaccia G. (2015). Adhesive anchors in high performance concrete. Mater. Struct..

[38] Tanaka Y., Himuro Y., Kainuma R., Sutou Y., Omori T., Ishida K. (2010). Ferrous Polycrystalline Shape-Memory Alloy Showing Huge Superelasticity. Science.

[39] Omori T., Ando K., Okano M., Xu X., Tanaka Y., Ohnuma I., Kainuma R., Ishida K. (2011). Superelastic Effect in Polycrystalline Ferrous Alloys. Science.

[40] Vollmer M., Krooß P., Karaman I., Niendorf T. (2017). On the effect of titanium on quenching sensitivity and pseudoelastic response in Fe-Mn-Al-Ni-base shape memory alloy. Scr. Mater..

[41] Abuzaid W., Wu Y., Sidharth R., Brenne F., Alkan S., Vollmer M., Krooß P., Niendorf T., Sehitoglu H. (2019). FeMnNiAl Iron-Based Shape Memory Alloy: Promises and Challenges. Shape Mem. Superelasticity.

[42] Vollmer M., Arold T., Kriegel M.J., Klemm V., Degener S., Freudenberger J., Niendorf T. (2019). Overcoming grain size limitations in Fe-base shape memory alloys through composition promoted abnormal grain growth. Nat. Commun..

